# Supplementary material for: Epidemiology and Outcome of Early-Onset Acute Kidney Injury and Recovery in Critically Ill COVID-19 Patients: A Retrospective Analysis
Source: Biomedicines. 2023 Mar 23;11(4):1001. doi: 10.3390/biomedicines11041001 (PMC10135848; doi:10.3390/biomedicines11041001)
Supplement: Supplementary file 1 [file biomedicines-11-01001-s001.zip › biomedicines-2242298-supplementary.pdf]

Supplementary data

Table S1. AKI prevalence and mortality rate in the ICU

Author	Country	Year	Number of patients	Prevalence of AKI				Mortality rate
				Global	***KDIGO 1***	***KDIGO 2***	***KDIGO 3***	
Lowe	UK	2021	81	44%	33%	22%	44%	25%
Lumlertgul	UK	2021	313	76.6%	20.1%	13.1%	43.5%	34% (90-day)
Lessa da Costa	Brazil	2021	102	55.9%	17.5%	15.7%	66.7%	33.3%
Alessandrini	Italy	2021	61	57.4%	17.1%	25.7%	57.2%	-
Candido de Almeida	Brazil	2021	278	71.2%	25.9%	8.9%	3.6%	39.8%
Arrestier	France	2022	70	64.3%	8.9%	11.1%	80%	51.1% (28-day)
Schaubroeck	Belgium	2022	1286	85.1%	12.1%	47.4%	25.7%	37.3% (28-day)
Geri	France	2021	379	52%	12%	25%	25%	26.4% (28-day)
Lumlertgul	UK	2022	772 (n = 316 - wave 1)<br>(n = 456 - wave 2)	76% (1)<br>51% (2)	27% (1)<br>53% (2)	17 (1)<br>17 (2)	56 (1)<br>30 (2)	29(1)<br>26(2)

Table S2: Characteristics of patients depending on occurrence of early onset and late AKI and requirement for renal replacement therapy.

	No AKI	EO-AKI	EO-AKI no RRT	EO-AKI RRT	Late-AKI	Late-AKI no RRT	Late-AKI RRT
Number	155	84	59	25	27	14	13
Age (years)	66.51 [59.53 ; 72.74]	73.71 [65.24 ; 76.88]	74.46 [64.28 ; 77.36]	72.81 [67.64 ; 75.45]	69.85 [60.48 ; 74.47]	71.41 [60.48 ; 76.19]	69.52 [66.4 ; 73.54]
Sex(male)	108 (69.68)	58 (69.05)	36 (61.02)	22 (88)	20 (74.07)	10 (71.43)	10 (76.92)
Body mass index > 30 kg/m²	64 (41.29)	41 (48.81)	24 (40.68)	17 (68)	12 (44.44)	7 (50)	5 (38.46)
Cardiac chronic failure	21 (13.55)	21 (25)	15 (25.42)	6 (24)	8 (29.63)	5 (35.71)	3 (23.08)
Respiratory chronic failure	12 (7.74)	3 (3.57)	2 (3.39)	1 (4)	2 (7.41)	1 (7.14)	1 (7.69)
Impaired immune system	21 (13.55)	19 (22.62)	15 (25.42)	4 (16)	3 (11.11)	1 (7.14)	2 (15.38)
Diabetes	18 (11.61)	17 (20.24)	9 (15.25)	8 (32)	5 (18.52)	4 (28.57)	1 (7.69)
Time from first symptoms to ICU admission	10 [8 ; 12]	8 [4.5 ; 10]	8 [4 ; 10]	7 [5 ; 11]	9 [7 ; 12]	9 [8 ; 12]	9 [7 ; 11]
Steroids	137 (88.39)	68 (80.95)	50 (84.75)	18 (72)	21 (77.78)	12 (85.71)	9 (69.23)
Antimicrobial therapy	42 (27.63)	40 (47.62)	28 (47.46)	12 (48)	11 (40.74)	6 (42.86)	5 (38.46)
SAPS II	34 [28 ; 41]	42 [33 ; 50]	41 [32 ; 48]	46 [34 ; 54]	35 [27 ; 39]	32 [29 ; 39]	38 [27 ; 41]
SOFA	4 [3 ; 5]	5 [4 ; 9]	5 [4 ; 8]	7 [5 ; 12]	5 [3 ; 6]	3.5 [3 ; 5]	5 [5 ; 6]
PaO2/FiO2	100 [72 ; 271]	90 [67 ; 190]	98 [70 ; 223]	79 [59 ; 122]	92 [62 ; 207]	108 [70 ; 221]	86 [55 ; 138]
Vasopressors	5 (3.23)	25 (29.76)	13 (22.03)	12 (48)	1 (3.7)	1 (7.14)	0
Invasive mechanical ventilation	6 (3.87)	22 (26.19)	11 (18.64)	11 (44)	2 (7.41)	2 (14.29)	0
RRT	0	3 (3.57)	0	3 (12)	0	0	0
Bacterial pulmonary co-infection	10 (6.58)	6 (7.14)	2 (3.39)	4 (16)	2 (7.41)	2 (14.29)	0
During ICU stay							
Vasopressors	8 (5.16)	52 (61.9)	27 (45.76)	25 (100)	25 (92.59)	12 (85.71)	13 (100)
Invasive mecanical ventiltaion	10 (6.45)	53 (63.1)	28 (47.46)	25 (100)	25 (92.59)	12 (85.71)	13 (100)
VAP	3 (1.97)	22 (26.19)	13 (22.03)	9 (36)	10 (37.04)	6 (42.86)	4 (30.77)
RRT	0	25 (29.76)	0	25 (100)	13 (48.15)	0	13 (100)
ICU LOS (days)	5 [4 ; 8]	10.5 [7 ; 16]	9 [6 ; 14]	16 [10 ; 21]	19 [15 ; 33]	18 [15 ; 31]	28 [17 ; 33]
ICU death	13 (8.39)	47 (55.95)	27 (45.76)	20 (80)	19 (70.37)	10 (71.43)	9 (69.23)
Hospital LOS (days)	13 [9 ; 20]	15 [9.5 ; 22.5]	15 [9 ; 23]	16 [12 ; 21]	22 [16 ; 46]	20.5 [16 ; 46]	28 [17 ; 38]
Hospital death	17 (10.97)	52 (61.9)	31 (52.54)	21 (84)	21 (77.78)	10 (71.43)	11 (84.62)
Death at 3 month	19 (13.29)	49 (64.47)	28 (53.85)	21 (87.5)	19 (76)	10 (71.43)	9 (81.82)

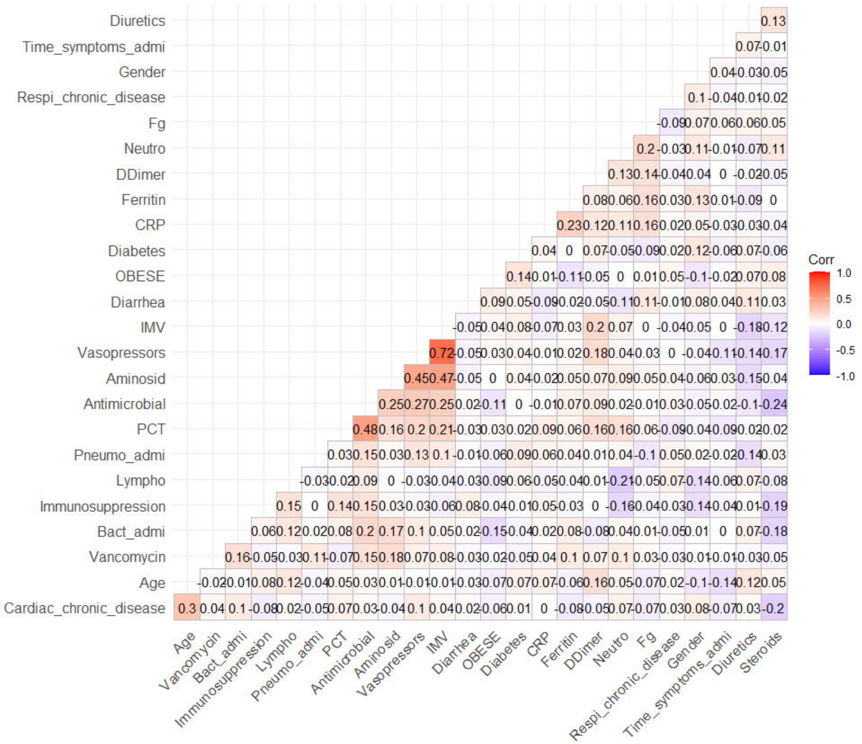

<u>Figure S1: correlation coefficient between covariates on admission</u>

Fg: fibrinogen; Neutro: Neutrophils; CRP: C-reactive protein; IMV: Invasive mechanical ventilation; PCT: procalcitonin; Pneumo_admi: bacterial respiratory infection on admission; Lympho: Lymphocytes; bact_admi: septicemia on admission

Table S3: Factors associated with occurrence of Early-Onset Acute Kindey Injury, uni and then multivariate logistic regression analyses.

		Univariate			Multivariate	
	OR	ORIC 95%	pvalue	OR	ORIC 95%	pvalue
**Age, 50-60 yrs**	1.29	[0.35 ; 4.77]	0.76	0.98	[0.24 ; 3.93]	0.59
**Age 60 -70 yrs**	1.03	[0.3 ; 3.48]	0.23	0.79	[0.22 ; 2.86]	0.16
**Age > 70 yrs**	3.07	[0.97 ; 9.73]	<.01	2.51	[0.74 ; 8.45]	<0.01
**Age < 50 yrs**	1		<0.01	1		<0.01
**Sex, men**	0.94	[0.54 ; 1.65]	0.83			
**BMI > 30 kg/m²**	1.33	[0.79 ; 2.24]	0.28			
**Cardiovascular disease**	1.76	[0.93 ; 3.31]	0.08			
**Chronic respiratory disease**	0.44	[0.12 ; 1.59]	0.21			
**Impaired immune system**[1]	1.93	[0.99 ; 3.75]	0.05			
**Diabetes mellitus**	1.75	[0.88 ; 3.49]	0.11			
**Time from first symptoms to ICU admission (days)**	0.42	[0.23 ; 0.75]	<0.01	0.42	[0.22 ; 0.81]	<0.01
**Diarrhea**	0.81	[0.28 ; 2.35]	0.70			
**Lopinavir-ritonavir**	2.19	[0.53 ; 8.97]	0.28			
**Remdesivir**	0.48	[0.22 ; 1.04]	0.06			
**Steroids**	0.65	[0.32 ; 1.29]	0.22			
**Tocilizumab**	0.51	[0.19 ; 1.42]	0.20			
**Antimicrobial therapy**	2.16	[1.27 ; 3.69]	<0.01			
**Aminoglycosides**	18.74	[2.3 ; 152.39]	<0.01			
**Vancomycin**	1.07	[0.1 ; 11.93]	0.96			
**Diuretics**	0.96	[0.52 ; 1.79]	0.91			
**PaO2/FiO2 200 – 300**	2.27	[0.78 ; 6.58]	0.24			
**PaO2/FiO2 100 – 200**	1.39	[0.56 ; 3.48]	0.73			
**PaO2/FiO2 < 100**	1.74	[0.79 ; 3.81]	0.54			
**PaO2/FiO2 > 400**	1		0.41			
**Vasopressors**	12.43	[4.86 ; 31.77]	<0.01			
**Invasive mechanical ventilation**	7.72	[3.27 ; 18.23]	<0.01			
**Pulmonary bacterial co-infection**	1.07	[0.39 ; 2.96]	0.90			
**Bacteriemia on admission**	3.98	[1.13 ; 13.98]	0.03			
**Neutrophils > 6 G/L**	1.19	[0.71 ; 2]	0.51			
**Lymphocytes < 0.6 G/L**	1.22	[0.72 ; 2.04]	0.46			
**C-reactive protein > 100 mg/L**	1.07	[0.64 ; 1.8]	0.79			
**DDimers > 1500 µg/L**	1.17	[0.68 ; 1.98]	0.57			
**Fibrinogen > 7 g/L**	0.78	[0.47 ; 1.31]	0.35			
**Ferritin > 1000 ng/mL**	0.99	[0.59 ; 1.65]	0.96			
**PCT > 0.5 g/L**	3.77	[2.14 ; 6.66]	<0.01	3.25	[1.76 ; 6.01]	<0.01

AKI, Acute kidney injury; BMI, body mass index; ICU, intensive care unit.

[1] Aplasia (lymphocytes < 1000/mm3); or corticosteroids (if treatment duration >1 month or if treatment amount >2mg/kg regardless of duration); or HIV (positive serology); AIDS (positive HIV serology and clinical complications: pneumocystis pneumonia, Kaposi's sarcoma, tuberculosis, toxoplasmosis.

Table S4: Factors associated with renal recovery after Early-Onset Acute Kidney Injury, uni and then sub distribution, survival analyses.

		Univariate			Multivariate	
**Variable**	SubHR	Sub HRIC 95%	pvalue	SubHR	Sub HRIC 95%	pvalue
**Age, 50-60 yrs**	0.29	[0.1 ; 0.8]	0.02			
**Age 60 -70 yrs**	0.23	[0.09 ; 0.61]	<0.01			
**Age > 70 yrs**	0.33	[0.16 ; 0.72]	0.01			
**Age < 50 yrs**	1		0.02			
**Sex, men**	0.91	[0.57 ; 1.47]	0.71			
**BMI > 30 kg/m²**	0.88	[0.56 ; 1.38]	0.58			
**Cardiovascular disease**	1.32	[0.8 ; 2.17]	0.28			
**Chronic respiratory disease**	0.84	[0.24 ; 2.97]	0.79			
**Impaired immune system**[1]	0.99	[0.58 ; 1.68]	0.96			
**Diabetes mellitus**	0.95	[0.48 ; 1.91]	0.90			
**Time from first symptoms to ICU admission (days)**	0.89	[0.51 ; 1.54]	0.68			
**Diarrhea**	1.09	[0.5 ; 2.38]	0.84			
**Lopinavir-ritonavir**	0.21	[0.03 ; 1.34]	0.10			
**Remdesivir**	1.07	[0.56 ; 2.06]	0.83			
**Steroids**	1.65	[0.83 ; 3.29]	0.15			
**Tocilizumab**	1.39	[0.51 ; 3.82]	0.52			
**Antimicrobial therapy**	0.85	[0.54 ; 1.33]	0.48			
**Aminoglycosides**	0.49	[0.19 ; 1.23]	0.13			
**Vancomycin**	0.91	[0.7 ; 1.17]	0.45			
**Diuretics**	0.65	[0.39 ; 1.09]	0.10	0.39	[0.26 ; 0.6]	<0.01
**PaO2/FiO2 200 – 300**	1.07	[0.43 ; 2.63]	0.89			
**PaO2/FiO2 100 – 200**	0.70	[0.31 ; 1.56]	0.38			
**PaO2/FiO2 < 100**	0.87	[0.43 ; 1.75]	0.69			
**PaO2/FiO2 > 400**	1		0.72			
**Vasopressors**	0.57	[0.32 ; 1.03]	0.06			
**Invasive mechanical ventilation**	0.76	[0.43 ; 1.36]	0.36			
**Renal replacement therapy**	0.27	[0.05 ; 1.4]	0.12			
**Pulmonary bacterial co-infection**	0.76	[0.31 ; 1.84]	0.54			
**Bacteriemia on admission**	0.85	[0.4 ; 1.8]	0.67			
**Neutrophils > 6 G/L**	1.02	[0.64 ; 1.62]	0.93			
**Lymphocytes < 0.6 G/L**	1.14	[0.73 ; 1.78]	0.57			
**C-reactive protein > 100 mg/L**	1.34	[0.86 ; 2.1]	0.20			
**DDimers > 1500 µg/L**	1.05	[0.64 ; 1.73]	0.84			
**Fibrinogen > 7 g/L**	1.10	[0.7 ; 1.73]	0.67			
**Ferritin > 1000 ng/mL**	1.06	[0.68 ; 1.67]	0.80			
**PCT > 0.5 g/L**	0.92	[0.59 ; 1.45]	0.73			
**KDIGO stage 2**	0.36	[0.19 ; 0.68]	<0.01	0.32	[0.17 ; 0.57]	<0.01
**KDIGO stage 3**	0.14	[0.07 ; 0.28]	<0.01	0.11	[0.05 ; 0.22]	<0.01
**KDIGO stage 1**	1		<0.01	1		<0.01

AKI, Acute kidney injury; BMI, body mass index; ICU, intensive care unit.

[1] Aplasia (lymphocytes < 1000/mm3); or corticosteroids (if treatment duration >1 month or if treatment amount >2mg/kg regardless of duration); or HIV (positive serology); AIDS (positive HIV serology and clinical complications: pneumocystis pneumonia, Kaposi's sarcoma, tuberculosis, toxoplasmosis
